# Incidence of Mast Cells in Oral Squamous Cell Carcinoma: A Short Study

**DOI:** 10.1155/2014/614291

**Published:** 2014-01-23

**Authors:** A. Anuradha, B. Kiran Kumar Naik, G. Vijay Srinivas, Ramisetty Sabitha Devi, H. K. Puneet

**Affiliations:** ^1^Department of Oral Pathology, St Joseph Dental College & Hospital, Duggirala, Eluru 534003, India; ^2^Anuradha ENT Hospital, D/No-10/200, Eluru Road, Gudivada, Krishna District, Andhra Pradesh 521301, India

## Abstract

Mast cells are regarded as complex and multifunctional cells, playing a significant role in immunopathology and a substantial role in tumor angiogenesis. Angiogenesis is a complex process that is tightly regulated by various growth factors in which mast cells act directly by releasing angiogenic factors and henceforth promoting tumor growth and metastasis. The aim of this study is to evaluate the number of mast cells in tissue sections of oral squamous cell carcinoma (OSCC) in comparison with normal mucosa. A total of 40 cases (20 OSCC and 20 normal mucosa) were stained with 1% toluidine blue and the quantitative analysis was done by using light microscope under 400x magnification. A significant increase in the mast cell count was observed in the sections of OSCC when compared to normal mucosa suggesting their contributing role in tumor growth and progression.

## 1. Introduction

Mast cells are normally present in small numbers in the connective tissue of all organs and more particularly (around blood vessels and nerves) in the dermal layer of skin, ranging from 5 to 15 *μ*m in diameter, and in histologic sections often appear ovoid, tadpole, or spindle shaped cells with cytoplasmic granules of 0.2 to 0.5 cm in size. They exert their influence locally and systemically by releasing a variety of potent mediators like histamine, leukotrienes, and cytokines through degranulation and cause neovascularization by producing angiogenic mediators such as fibroblast growth factor (FGF), transforming growth factor-*β* (TGF), tumor necrosis factor-*α* (TNF), and vascular endothelial growth factor (VEGF). They occur in various pathological states and also in some benign and malignant tumors. An attempt has been made to quantitatively estimate the number of mast cells in OSCC and to signify their role in tumor growth and progression [1–3].

## 2. Materials and Methods

20 paraffin embedded specimens of OSCC of age groups from 30–80 years with no systemic illness but having the habit of smoking and alcohol consumption for a period of 5–10 years were retrieved from the archives of the department of oral pathology, among which seven cases were of well differentiated squamous cell carcinoma (*n* = 7; females = 4, males = 3) and 13 were moderately differentiated squamous cell carcinoma (*n* = 13; 4 = females, 9 = males). 20 normal oral mucosal biopsies from age groups of 15–20 years with no systemic illness were obtained from 20 adult patients undergoing extraction for orthodontic treatment. Serial sections of 5 *μ*m thickness were made from paraffin embedded tissue blocks using semiautomatic microtome (Thermo Scientific Microm HM340E). All the sections were stained with 1% toluidine blue as per Churukian and Schenk method [4].

**Figure 1:**
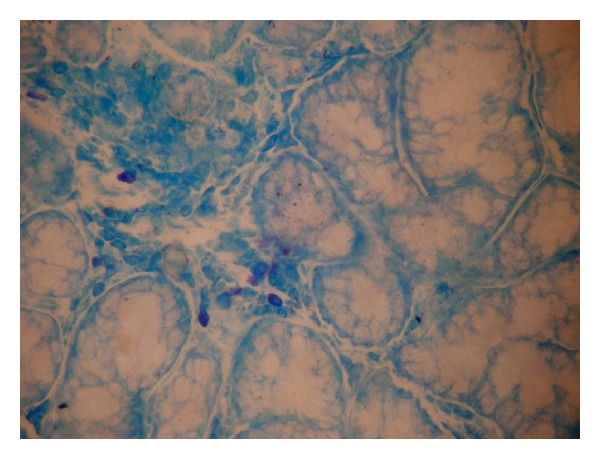
Mast cells in controls (40x).

**Figure 2:**
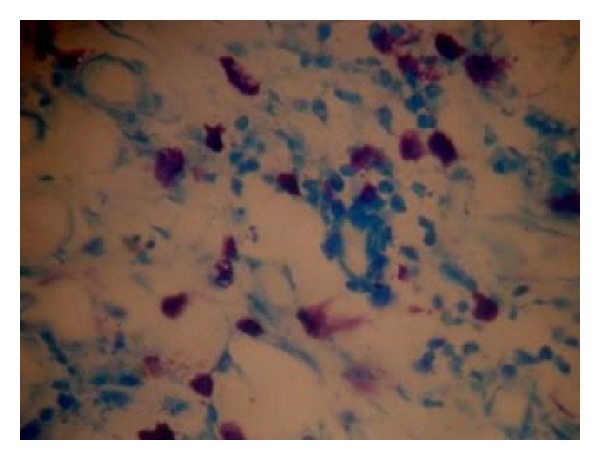
Mast cells in oral squamous cell carcinoma (40x).

Mast cells were counted under light microscope at a magnification of 400x in a *Z*-pattern from left to right and the data obtained was statistically analyzed using ANOVA. (Figures 1 and 2).

## 3. Criteria to Identify the Mast Cells

Mast cell granules are purplish red and the nuclei of mast cells appear sky blue in color. Toluidine blue stains the mast cell granules metachromatically due to its reaction with sulphated mucopolysaccharides [1].

## 4. Result

**Figure 3:**
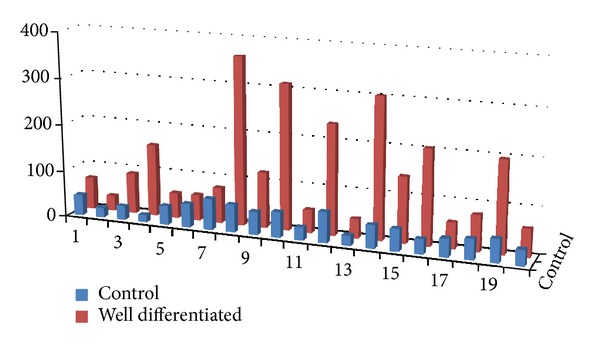
Bar graph showing increase of mast cell count in OSCC when compared to controls.

**Figure 4:**
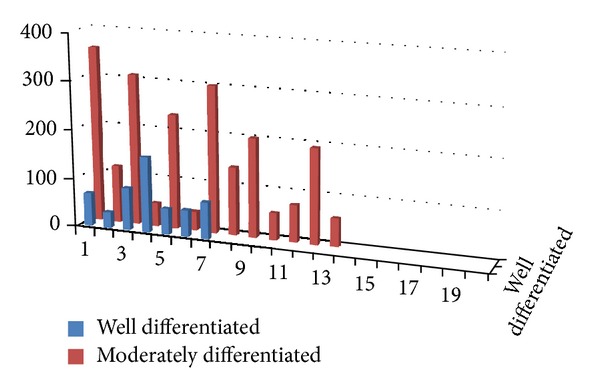
Bar graph showing mast cell count between well and moderately differentiated OSCC.

A highly significant (*P* = 0.00) threefold increase in the average number of mast cells/slide was observed in OSCC when compared to controls (Table 1, Figure 3). No statistical difference (*P* = 0.058) was found in the average number of mast cells/slide when moderately and well-differentiated OSCC were compared. (Table 2, Figure 4). Average count of mast cells/microscopic field under 400x magnifications was found to be 3 in OSCC and 2 in controls.

## 5. Discussion

Paul Ehrlich in 1877 discovered a granular cell of loose connective tissue and named it as “Mastzellen”—a well fed cell. Studies on mast cells in normal and various pathologic conditions have shown them to be complex, well-engineered, and multifunctional cells playing an essential role in acquired and innate immunity. They take origin from multipotent CD 34+ precursor in the bone marrow, later circulate in the peripheral blood as agranular monocytic cell, and then migrate into tissues, assuming their typical granular morphology from their immature state. They are normally distributed throughout the connective tissue, adjacent to blood or lymphatic vessels, and near or within peripheral nerves. They are numerous especially beneath the epithelial surfaces of the skin, in the respiratory system, gastrointestinal and genitourinary tracts. Many of the mediators are stored within cytoplasmic granules and these include preformed mediators like histamine, heparin, and tryptase; lipid derived mediators like leukotriene's B4 (LTB4), LTC4, LTD4, and LTE4; proinflammatory cytokines like TNF-*α*, IL-1; mitogenic cytokines like IL-3, IL-5; and immunomodulatory cytokines like IL-4, IL-10, and serotonin and other mediators are produced at the time of mast cell stimulation such as IL-1 [5, 6].

Mast cells have been considered the tissue equivalent of the circulating basophils though they arise from a common precursor cell in the bone marrow. There is no evidence that mature basophils are able to differentiate into mast cells. The two cell types are readily distinguished by their morphology on light microscopy and the presence of chloroacetate esterase activity in mast cells. They have been studied in normal gingiva, chronic inflammatory gingivitis, desquamative gingivitis, lichen planus, oral submucous fibrosis (OSMF), and OSCC. They exhibit phenotypic plasticity and variation in the mast cell mediators with the change in the microenvironment. Volumes of literature speak about their role in the inflammatory reactions and neovascularization. In some malignancies, large numbers of mast cells were detected before the occurrence of neovascularization [2, 7].

In carcinogenesis, angiogenic regulation is biphasic. In the premalignant early phase of hyperplasia and dysplasia, infiltrating mast cells degranulate and activate dermal fibroblasts which intensify angiogenesis. They even activate progelatinase B (a member of the matrix metalloproteinase (MMP) family) which is involved in both extracellular remodeling [8] and regulation of angiogenesis [9]. The mast cells activate and progressively intensify angiogenesis by releasing sequestered angiogenic activators. As neoplastic progression proceeds, angiogenic growth factor gene expression is upregulated in the cancer cells, marking progression to the second cancer phase, wherein the tumor cells control their angiogenic phenotype directly instead of depending on the manipulation of inflammatory cells to indirectly affect neovascularization [10–14]. This implies that mast cells have significant role in the early stages of cancer progression and increase in mast cells is observed in the initial stages whereas, in second cancer phase, the mast cells decrease because the tumor cells are not dependent on them anymore for the neovascularization effect and this could be the reason for the increase in the mast cells in moderate when compared to well-differentiated OSCC in our study and also might be due to interobserver mystification in classifying the well- and moderate differentiated forms of OSCC when compared to poorly differentiated.

Though many studies have proven the presence and significance of mast cells, their mediated angiogenesis is complex and not completely understood. In harmony with the literature, our study also showed a high mast cell count in OSCC than in control tissue indicating their supportive role in tumor progression and metastasis.

## 6. Conclusion

This study reveals that there is a definitive increase in mast cells count when compared to normal mucosa substantiating their contributing role in tumor progression.

## Figures and Tables

**Table 1:** Comparison of the average mast cell count in between OSCC and control group using ANOVA test.

Group	Sample size (*n*)	Average number of mast cells/slide	*P* value
OSCC	20	135.90	0.000
Controls	20	42.85	

**Table 2:** Comparison of the average mast cell count within the OSCC group using ANOVA test.

Group	Sample size (*n*)	Average number of mast cells/slide	*P* value
Well dif	7	77.86	0.058
Mod diff	13	167.15	

## References

[1] Stone KD, Prussin C, Metcalfe DD (2010). IgE, mast cells, basophils, and eosinophils. *Journal of Allergy and Clinical Immunology*.

[2] Theoharides TC, Conti P (2004). Mast cells: the JEKYLL and HYDE of tumor growth. *Trends in Immunology*.

[3] Pansrikaew P, Cheewakriangkrai C, Taweevisit M, Khunamornpong S, Siriaunkgul S (2010). Correlation of mast cell density, tumor angiogenesis, and clinical outcomes in patients with endometrioid endometrial cancer. *Asian Pacific Journal of Cancer Prevention*.

[4] Sudhakar R, Ramesh V, Balamurali PD, Nirima O, Premalatha B (2005). Karthikshree. Incidence of mast cells in oral inflammatory lesions: a pilot study. *Journal of Oral and Maxillofacial Pathology*.

[5] Ankle MR, Alka DK, Nayak R (2007). Mast cells are increased in leukoplakia, oral submucous fibrosis, oral lichen planus and oral squamous cell carcinoma. *Journal of Oral and Maxillofacial Pathology*.

[6] Kamal R, Dahiya P, Palaskar S, Shetty VP (2011). Comparative analysis of mast cell count in normal oral mucosa and oral pyogenic granuloma. *Journal of Clinical and Experimental Dentistry*.

[7] Mohtasham N, Babakoohi S, Nejad JS (2010). Mast cell density and angiogenesis in oral dysplastic epithelium and low- and high-grade oral squamous cell carcinoma. *Acta Odontologica Scandinavica*.

[8] Coussens LM, Werb Z (1996). Matrix metalloproteinases and the development of cancer. *Chemistry and Biology*.

[9] Vu TH, Shipley JM, Bergers G (1998). MMP-9/gelatinase B is a key regulator of growth plate angiogenesis and apoptosis of hypetrophic chondrocytes. *Cell*.

[10] Coussens LM, Raymond WW, Bergers G (1999). Inflammatory mast cells up-regulate angiogenesis during squamous epithelial carcinogenesis. *Genes and Development*.

[11] Elpek GÖ, Gelen T, Aksoy NH (2001). The prognostic relevance of angiogenesis and mast cells in squamous cell carcinoma of the oesophagus. *Journal of Clinical Pathology*.

[12] Polverini PJ (1995). The pathophysiology of angiogenesis. *Critical Reviews in Oral Biology and Medicine*.

[13] Ribatti D, Crivellato E (2009). Chapter 4 the controversial role of mast cells in tumor growth. *International Review of Cell and Molecular Biology*.

[14] Rakesh S, Vidya MJRB, Savithri VV (2012). Analysis of mast cell counts in oral leukoplakia. *Oral & Maxillofacial Pathology Journal*.

